# Prevention and Management of Infections in Lung Transplant Recipients

**DOI:** 10.3390/jcm13010011

**Published:** 2023-12-19

**Authors:** Anum Fayyaz, Mohammed Raja, Yoichiro Natori

**Affiliations:** Miami Transplant Institute, Jackson Health System, Division of Infectious Disease, Department of Clinical Medicine, University of Miami Miller School of Medicine, Miami, FL 33136, USA; anum.fayyaz@jhsmiami.org (A.F.); mxr1829@miami.edu (M.R.)

**Keywords:** lung transplant recipients, immunosuppression, antibiotics, infections

## Abstract

Anti-rejection medications are essential in preventing organ rejection amongst solid organ transplant recipients; however, these agents also cause profound immunosuppression, predisposing lung transplant recipients (LTRs) to infectious complications. The timely management including prevention, diagnosis, and treatment of such infectious complications is vital to prevent significant morbidity and mortality in solid organ transplant recipients and allograft dysfunction. LTRs are inundated with microbes that may be recognized as commensals in hosts with intact immune systems. Bacterial infections are the most common ones, followed by viral pathogens. Indications of a brewing infectious process may be subtle. Hence, the importance of adapting vigilance around isolated hints through symptomatology and signs is pivotal. Signals to suggest an infectious process, such as fever and leukocytosis, may be dampened by immunosuppressive agents. One must also be vigilant about drug interactions of antibiotics and immunosuppressive agents. Treatment of infections can become challenging, as antimicrobials can interact with immunosuppressive agents, and antimicrobial resistance can surge under antimicrobial pressure. Transplant infectious disease physicians work in concert with transplant teams to obtain specimens for diagnostic testing and follow through with source control when possible. This heavily impacts medical decisions and fosters a multidisciplinary approach in management. Furthermore, the reduction of immunosuppression, although it augments the risk of allograft rejection, is as crucial as the initiation of appropriate antimicrobials when it comes to the management of infections.

## 1. Risk of Infection and the Timeline of Predisposition to Infection

The risk of acquiring infections post solid organ transplantation depends on the epidemiologic risks and the host’s overall net state of immune suppression [1]. Epidemiologic risks predispose to bacterial, viral, fungal, and parasitic infections [2]. Infections can be community-acquired and nosocomial, depending on the host’s defense mechanism and time since transplant. Intense immune dysfunction predisposes to infection with a more significant disease burden and severity than that in non-immunocompromised individuals [1,3]. Although bronchoscopy protocols vary by institution, they play a significant role in the timely diagnosis of infections as well as rejection [4]. Understanding predisposing risk factors to infections in LTR patients is not straightforward [3,5]. We are now learning that the interaction between the microbiome and the immune system influences immune dysregulation and graft survival. Our perception regarding the microbiome has been elaborated recently, and interestingly, the microbiome in LTRs is even more complex. The microbiome of a transplant recipient is a function of prior colonization of mucosal surfaces, the allograft microbiome, and pathogens derived from the community or nosocomial exposures [6]. Immunosuppression, infections, changing antimicrobials, preexisting comorbidities, and breaches in mucosal barriers due to surgery all contribute to alterations in the microbial milieu of an immunocompromised host [6,7]. Further research will determine the impact of this understanding on the clinical management of LTRs.

Epidemiology influences the acquisition of new infections, reactivation of quiescent infections, or exacerbation of untreated infections. This understanding helps us decide on appropriate donor and recipient screening for infections before transplantation and initiation of immunosuppression [7]. Please view Table 1 for common tests in donors. Although certain donors are considered high risk by the Public Health Service (PHS) we routinely perform *HIV* and *hepatitis B* and *C* screening in all donors. Devising appropriate preemptive or prophylaxis strategies against anticipated pathogens also highlights the cognitive acumen practiced while managing LTRs.

## 2. Bacterial Infection in LTRs

The most frequent infectious complications following LT are bacterial infections, about 50% of which predominantly present as pneumonia [2]. Pneumonia can be acute or chronic, community-acquired, hospital-acquired, or due to opportunistic pathogens in this patient population. Several risk factors can result in pneumonia, including underlying medical comorbidities such as cystic fibrosis (CF) predisposing to colonization by *multidrug-resistant (MDR) bacteria*, cardiovascular disease, and post-transplant requirement for mechanical ventilation. The risk of infection is an interaction of two pivotal factors: epidemiologic risk factors and a net state of immunity [2,8].

## 3. Pre-Lung Transplant

A comprehensive understanding of the recipient is pivotal to improving outcomes post-lung transplant [2,9]. This entails obtaining a thorough history that considers existing comorbidities, previous infections/colonization, and either resolution of infection or a controlled state before transplant. Obtaining extensive social history to understand environmental and epidemiological risk factors that may predispose to infections is also paramount. Assessment of colonization by various pathogens is essential to guide prophylactic therapy, time to transplant, and an indication of a double lung transplant. Although protocols for perioperative antimicrobial prophylaxis vary across centers, generally, broad-spectrum antimicrobials are utilized [10]. Poor post-transplant outcomes have been described due to prior colonization and previous or active infection, especially with MDR pathogens. Colonization or infection secondary to *Burkholderia cepacia* complex and *Mycobacterium abscessus* result in contraindication to a single lung transplant, and even for a double lung transplant, the risk is increased [11]. Screening with rectal swabs for colonization of specific MDR pathogens, e.g., vancomycin-resistant *Enterococcus* (VRE), *Klebsiella pneumoniae* carbapenemase (KPC), and nasal swabs for MRSA prior to transplantation is suggested in certain patients; however, this is not a universal practice [12].

Post-lung transplant infections in immunosuppressed hosts can be distributed over a timeline starting from the time of transplant (Table 2). This is due to changing risk factors starting from surgery, progressive immunosuppression, predisposition to the emergence of latent infections, and community exposures. Operative intervention results in anatomical breach in sterility and deprives LTRs of primary defense mechanisms. This predisposes them to hospital-acquired infections, aspiration pneumonia, central line-associated bloodstream infections (CLABSI), and urinary catheter-associated infections. The proposed timeline represents three overlapping periods of risk: within the first month of transplantation, 1 to 6 months post-transplantation, and more than 6 months after transplantation [13].

## 4. 0–1 Month Post LTR 

LTRs are at the highest risk of infections in the first month post-transplant. While evaluating an LTR, it is helpful to consider possible predisposing factors during the first month post-transplant [14]. These factors include surgical complications, donor-derived infections, untreated or partially treated recipient infections, and nosocomial infections [15]. Infections early in transplant often reflect surgical complications (e.g., anastomotic complications or bleeding) [16]. There has also been a consideration in case reports and case series of hyperammonemia syndrome (HS) in lung transplant recipients in association with *Mycoplasma hominis* and ureaplasma. Although the management is far more complex, we do consider antimicrobial management targeting these pathogens for HS [17].

The presence of fever by itself may not necessarily be a sign of an infectious process. Non-infectious complications such as organ rejection and blood transfusion can also cause fever. We are looking into refined diagnostics, such as imaging modalities, that may help differentiate infection from rejection. However, a provider must be vigilant to consider all possibilities within the clinical context and pursue a thorough infection risk evaluation. Timely antimicrobial initiation and source control are important in these immune-suppressed hosts. Pretransplant immunosuppression is a risk factor for early opportunistic infections during this period, even though it is less likely. Otherwise, opportunistic infections are less common during the first month post-transplant [18].

Pneumonia secondary to *non-tuberculous mycobacterium (NTM)* is associated with high mortality in LTRs. The majority of transplant centers screen for *NTM* colonization before transplant [19]. This practice should determine candidacy for a double/single lung transplant. We recommend that *rapid-growing mycobacterium (RGM)* pretransplant colonized patients undergo double lung transplantation. Post-transplant patients are not routinely evaluated for *NTM colonization;* however, LTRs do develop post-transplant NTM pneumonia. The majority of lung transplant centers require respiratory culture negativity prior to transplant. On the other hand, routine post-transplant bronchoscopies help in the detection of NTM, and timely management can result in improved outcomes [4,19,20,21].

## 5. 1–6 Months and after 6 Months Post LT

Six months post-transplant is a prime window for acquiring opportunistic pathogens [9]. Post-transplant antimicrobial prophylaxis has modified the risk and improved transplant outcomes [3,9]. When initiating empiric antimicrobial therapy, pathogen and host factors must be considered, such as bacterial colonization or previous infections with *MDR pathogens*, local epidemiology and antibiogram, the patient’s allergy profile, and drug interactions. The common pathogens and located infections are summarized in Table 2.

## 6. Viral Infections in LTR

After bacterial infections, viruses are the second leading cause of infections in LTRs. The necessity of an intact cellular immune system to limit morbidity associated with viral respiratory illness is pivotal [22]. The impaired T-cell immunity in the LTR population facilitates the acquisition of viruses and delays viral clearance, contributing to the disease process and severity [22,23]. The risk of acquisition of viral pathogens peaks with immunosuppression and subsides with the maintenance of immune suppression. However, LTRs are at high risk of community-acquired respiratory virus infections, which may even predispose them to superimposed bacterial infections.

It is well known that *Cytomegalovirus (CMV)* attains latency and is kept at bay by our surveilling immune system. On top of donor-derived *CMV* infection, *CMV* can reactivate when the cellular immunity is impaired, usually one month into LT. This risk is maintained through months post-transplant [24]. However, delayed *CMV* infection is a known risk in high-risk patient populations [25]. The interplay of factors discussed above plays a similar role in predisposing patients to viral infections. Screening for viruses pre-transplant allows for preemptive measures to decrease the risk of reactivation or acquisition (Table 3). Herpes viruses such as *CMV* and *EBV* can also be surrogate markers of immunity. Detection of these markers in the serum suggests immunosuppression and predisposition to other infections. Other viral etiologies to consider, although less commonly studied, include hepatotropic viruses, *West Nile virus (WNV)*, *Human T-lymphotropic virus (HTLV)-1/2, rabies, Zika virus, and lymphocytic choriomeningitis virus (LCMV)*.

## 7. Pre-LT Recipient and Pre-LT Donor Screening

Although there may be epidemiologic risk-related variations, most donors and recipients undergo screening for these viruses: herpesviruses *(Herpes simplex virus HSV-1 and HSV-2, Epstein–Barr virus (EBV), CMV, Varicella Zoster virus (VZV)), hepatotropic viruses (HAV, HBV, HCV), human immunodeficiency virus (HIV), measles, mumps, and rubella (MMR)*. Transplantation is usually postponed in an acute viral infectious process (Table 3).

LTRs are predisposed to the seasonality of community-acquired viruses [26]. Due to impaired defense mechanisms in LTRs, upper respiratory viral infection progresses to lower respiratory infection and contributes to morbidity and mortality. Prevention of community-acquired viruses through vaccination is highly recommended [27]. Influenza seasonal vaccine is recommended to patients. If patients acquire the Influenza virus, neuraminidase inhibitors are administered for more than 5 days due to prolonged viral shedding in T-cell-depleted LTRs. The recently approved *respiratory syncytial virus (RSV)* vaccine is also supported and administered now. There is no definitive treatment for RSV infection in immunocompromised hosts [28]. Although inhaled ribavirin is utilized, there are no universal protocols for initiation and duration of treatment [29]. Palivizumab has been studied for the prevention of *RSV*, but the data come from pediatric patients, not from the transplant population [30]. Further vaccines should be given based on national and international guidelines. The International Society of Heart and Lung Transplantation (ISHLT), in the wake of the *SARS-CoV-2 (COVID-19)* pandemic, guided us through the management of LTRs [23,31]. If patients are exposed to *COVID-19* but are asymptomatic >7 days after exposure with two negative PCR tests performed 24 to 48 h apart, and if the patients are at high risk without a transplant, we agree to transplant. In asymptomatic patients with positive PCR tests, we suggest 14 days after diagnosis with two negative PCR tests at least 24 to 48 h apart before proceeding with a transplant. In previously symptomatic patients, following clinical resolution and at least 28 days from the onset of symptoms, with two negative PCR tests performed 24 to 48 h apart (including a respiratory sample of the lower airways), negative chest CT, and no other COVID-19-related organ damage, we agree to transplant.

*CMV* serological tests are sensitive and specific guides in determining the seropositivity or seronegativity of the donor and recipient. CMV donor positivity is a high risk for a *CMV* seronegative recipient regardless of the induction of immunosuppressive agents (Table 4). There are data to guide preemptive monitoring in high-risk liver transplant patients; due to logistic reasons, most centers prefer prophylaxis [32]. As of now, it is still unclear if we can generalize this data to LTRs (Table 3).

There are refined treatment options for *HBV, HCV, and HIV*; incidental detection of these pathogens may not delay transplantation but may result in a reassessment. Regarding *hepatitis B and C*, the guidelines have been modified for new therapeutics. If the donor has evidence of *hepatitis B* infection suggested by the presence of positive HBsAg or HBcAb IgM, then a risk vs. benefits discussion towards transplantation can be undertaken regardless of the recipient’s immunity to hepatitis B [25]. The indication of hepatitis B treatment is summarized in Table 5. Even in *HCV* screening, few variations in donors have arisen. If *HCV* naïve patients receive a lung transplant from an *HCV* viremic patient, treatment is suggested after the transplant [33].

## 8. Fungal Infections in LTRs

Among LTRs, 8.6% develop an invasive fungal infection within 1 year following transplant (Table 6) [34]. Unlike in other transplants, *Aspergillus* is predominant after lung transplant, whereas candidiasis is predominant in other transplants. Several factors contribute to survival following lung transplantation. These include donor selection techniques, surgical methods, and perioperative care. However, LTRs remain at risk of invasive fungal infections (IFIs) post-transplant. The most frequently encountered pathogens are outlined in Table 6. 

Underlying anatomic barriers, inadequacy due to operative intervention, and weakened physiologic defense barriers in combination with environmental exposure augment the risk of IFI [35,36,37]. Works in the literature have explored other risk factors such as airway ischemia, weakened innate and humoral immune responses, foreign objects or instrumentation such as bronchial stents, fungal colonization or past infection, as well as infection with pathogens that are immunomodulatory, such as *CMV* disease. All predispose the patient to IFI [35]. Mold-active agents would take precedence if the recipient is colonized with *Aspergillus* sp. 

## 9. Pre-LT

Screening for colonization must be selective and based on the host and epidemiologic risk factors. When we screen, neither serum 1,3-b-D-glucan nor Galactomannan are recommended, as both are not validated for screening purposes. On the other hand, serial sputum culture and bronchoalveolar lavage, when feasible, are traditional but still useful tools to detect mold colonization before transplant.

## 10. Post-LT

There is a center-based variance in the utilization of fungal prophylaxis. Most lung transplant centers in the U.S. follow a universal prophylaxis approach; some still apply preemptive methods. Although a meta-analysis supports that antifungal prophylaxis is functional against IFI, further refined studies are needed for a more comprehensive approach [36]. The documented time for prophylaxis is also variable, ranging anywhere from 3 to 12 months post-LTR. Inhaled amphotericin B is a prophylaxis agent; however, there is a lack of consensus on dosing and duration [34,38,39]. Triazoles such as voriconazole and posaconazole are also utilized for prophylaxis, as favored by the side-effect profile. Although Isavuconazole has been suggested to be non-inferior to voriconazole in the treatment of IFI, prophylaxis with Isavuconazole has limited data, especially in LTRs [39]. Echinocandins can be utilized but have restrictive coverage and miss out on molds. Prophylaxis against *Pneumocystis jirovecci* is taken care of by trimethoprim-sulfamethoxazole. In cases of intolerance or allergies, alternative options are available, such as Atovaquone and Pentamidine.

In a recent prospective multicenter study, the prevalence rate for IFI was 19.1 per 100 surgeries (95% CI 16.4–21.8%) within 6 months after lung transplant [34]. Patients who had IFI episodes had a predisposition to recurrence. Risk factors for the development of IFI in LTRs include ECMO, persistent ventilation beyond 48 h of transplant, the need for hemodialysis, low functional status, and a high lung allocation score [35].

Taking risk factors into account, each center should determine proper prevention methods, with either serial routine bronchoscopy or universal prophylaxis, or a combination of the two.

## 11. Parasitic Infections in LTR

Parasitic infections in LTRs are less common than other infectious etiologies [40]. Although parasitic infections are more endemic in tropical climates, interest in travel has increased the risk of parasitic infections in LTRs. Relatively common parasitic infections are *malaria, leishmaniasis, strongyloidiasis, toxoplasmosis*, and *schistosomiasis*. The risk of infection is further subclassified as new onset disease in travelers, as a donor-derived process or a reactivation of infection, the latter two depending on the timeline from transplant. It has been suggested that parasitic infections follow two predominant clinic presentations after lung transplant. Infection can either involve one organ or be a disseminated systemic process. Conducting a pre-transplant assessment and considering risk factors that may predispose to parasitic infections is suggested.

*Toxoplasmosis* has a global distribution. Infection can occur during reactivation and through transplanted organs and newly acquired diseases. In LTRs, *toxoplasmosis* is a rare entity. Transmission is observed in seronegative recipients who received grafts from seropositive donors. Disseminated disease and encephalitis are the most severe presentations of *toxoplasmosis*. Effective prophylaxis against toxoplasmosis is carried out by administering Trimethoprim-sulphamethoxazole [41]. Serology is obtained in patients with risk factors for acquiring *Strongyloides stercoralis*. Prophylactic Ivermectin is suggested in a divided dose if a patient is seropositive [42].

## 12. Future Directions in Lung Transplantation

Although we are far from our first lung transplant, we have miles to go. Work is underway to refine diagnostics such as advanced imaging techniques to differentiate infection from rejection [43]. There are advancements in molecular tests, such as next-generation sequencing; however, the sensitivity of such tests can result in over-treatment and compromise antimicrobial stewardship. Hence, integrated work in antimicrobial and microbiologic testing stewardship is warranted for better outcomes [44] Other fields of interest are host microbiome as well as biomarker discovery such as hyperammonemia to refine management. Transplant immunology such as CMV-specific cell-mediated immunity (CMV-CMI) is being investigated as a potential tool to help determine the host’s defense against CMV and guide management in SOT recipients, other than lung transplant. CMV-CMI is also being studied to determine the duration of primary prophylaxis and secondary prophylaxis, and to guide the initiation of treatment against low-level CMV replication [45].

## 13. Conclusions

This paper aimed to summarize infectious ailments in lung-transplanted patients. This document may help define the infection risk in LT hosts. Although our understanding of LTRs has increased recently, we require more refined studies to help guide the prevention, prophylaxis, and treatment of infections in this specific patient population.

## Figures and Tables

**Table 1:** Common Tests for Infections in Donors.

Bacteria	Blood Culture
	Sputum Culture
Vital	Hepatitis C Virus Nuclear Antigen Amplification
	Human Immune Deficiency Virus (HIV) Nuclear Antigen Amplification
	4th generation HIV
	Hepatitis B Core Antibody
	Hepatitis B Surface Antigen and Antibody
	Hepatitis B Nuclear Antigen Amplification
	Human T Lymphotropic Virus Nuclear Antigen Amplification and I/II Antibody
	Cytomegalovirus Serology IgG
	Epstein–Barr Virus (VCA) (IgG)
	Ebstein–Barr Virus (VCA) (IgM)
	Epstein–Barr Virus Nuclear Antigen
	West Nile Nuclear Antigen Amplification
	Nasopharyngeal Swab for SARS COVID-19
Fungal	Sputum Culture
Parasite	Toxoplasma (IgG)
	Chagas NAT
	Strongyloidiasis Serology

**Table 2:** Timeline of Common Post-transplant Infections.

Timeline of Common Post-Transplant Infections			
Source	<4 weeks	1–12 months	>12 months
	Nosocomial, related to surgery, donor or recipient-derived	Activation of latent infections, opportunistic infections	Community-derived infections
Bacteria	Bacterial infections are due to the following scenarios. Anastomotic leak, Clostridium dificille, line infection, wound infections, nosocomial pneumonia, urinary tract infections	Listeria, Nocardia, Mycobacterium	Community-derived infections
Viruses	Donor-derived viruses	Herpes group (CMV, EBV, HHV6, 8, HSV, VZV), Hepatitis viruses (HAV, HBV, HCV, HEV), Retroviruses (HIV, HTLV-1 and 2)	Community-acquired viruses, CMV, HPV, JC Polyoma Virus, PTLD
Fungi	Candida species	Aspergillus, endemic fungi, Mucor, Scedosporium, PCP	Aspergillus, Mucor, Scedosporium, Cryptococcus
Parasites	Less likely	*Leishmania,* *Toxoplasma gondii, Trypanosoma Cruzii,* *Strongyloides stercoralis*	*Strongyloides stercoralis*

**Table 3:** Screening for Viruses.

Screening for Viruses
Human immunodeficiency virus serology (ELISA) or fourth-generation ELISA
Hepatitis B (HBV) serologies including HBV surface antigen (or HepB NAT), core antibody, surface antibody, QNAT if +
Hepatitis C antibody, QNAT if +
Cytomegalovirus antibody
Epstein–Barr virus (EBV) antibody panel (EBV viral capsid antigen, +/− early antigen, and nuclear antigen antibody levels)
Measles, mumps, rubella serologies

**Table 4:** Post-LT Monitoring and Prevention.

CMV Serologic Status	Risk for Reactivation	Possible Regimen	Suggested Duration of Follow-Up
D+/R−	High Risk	>12 months of prophylaxis with valganciclovir	Monthly for 6 months after discontinuation of therapy
D+/R−	High Risk	>12 months of prophylaxis with valganciclovir	Monthly for 6 months after discontinuation of therapy
R+	Intermediate Risk	6–12 months	For symptoms (may monitor monthly for 3–6 months after therapy)
R+	Intermediate Risk	6–12 months	For symptoms (may monitor monthly for 3–6 months after therapy)
D−/R−	Low Risk	Targeting HSV/VZV with Acyclovir	Symptoms follow-up

**Table 5:** Indications for Hepatitis B Treatment in Lung Transplant Recipients.

Indications for Hepatitis B Treatment in Lung Transplant Recipient	
HbsAg-positive recipient	HBIG + oral antiviral therapy
Anti-HBc-positive/Anti-HBs-negative recipients	Oral antiviral therapy
Anti-HBc-negative/Anti-HBs-positive recipient	Oral antiviral therapy

**Table 6:** Common Fungal Pathogens in LTRs.

Most Common Fungal Pathogens in Lung Transplant Patients
Aspergillus
Invasive candidiasis
Cryptococcus
*Pneumocystis* jirovecii pneumonia
*Scedosporium* spp.
Fusarium

## References

[1] Avery R.K. (2006). Infections after lung transplantation. Semin. Respir. Crit. Care Med..

[2] Sims K.D., Blumberg E.A. (2011). Common infections in the lung transplant recipient. Clin. Chest Med..

[3] Yun J.H., Lee S.-O., Jo K.-W., Choi S.H., Lee J., Chae E.J., Do K.-H., Choi D.-K., Choi I.-C., Hong S.-B. (2015). Infections after lung transplantation: Time of occurrence, sites, and microbiologic etiologies. Korean J. Intern. Med..

[4] Wahidi M.M., Ernst A. (2004). The role of bronchoscopy in the management of lung transplant recipients. Respir. Care Clin. N. Am..

[5] Parada M.T., Alba A., Sepúlveda C. (2010). Early and late infections in lung transplantation patients. Transplant. Proc..

[6] Fishman J.A. (2017). Infection in organ transplantation. Am. J. Transplant..

[7] Humar A., Michaels M., AST ID Working Group on Infectious Disease Monitoring (2006). American Society of transplantation recommendations for screening, monitoring, and reporting of infectious complications in immunosuppression trials in recipients of organ transplantation. Am. J. Transplant..

[8] Remund K.F., Best M., Egan J.J. (2009). Infections relevant to lung transplantation. Proc. Am. Thorac. Soc..

[9] Malinis M., Boucher H.W., AST Infectious Diseases Community of Practice (2019). Screening of donor and candidate before solid organ transplantation guidelines from the American society of Transplantation Infectious Diseases Community of Practice. Clin. Transplant..

[10] Chang S.H., Krupnick A.S. (2012). Perioperative antibiotics in thoracic surgery. Thorac. Surg. Clin..

[11] Kramer M.R., Stoehr C., Lewiston N.J., Starnes V.A., Theodore J. (1992). Trimethoprim-sulfamethoxazole prophylaxis for pneumocystis carinii infections in heart-lung and lung transplantation—How effective and for how long?. Transplantation..

[12] Shoham S., Shah P.D. (2013). Impact of multidrug-resistant organisms on patients considered for lung transplantation. Infect. Dis. Clin. N. Am..

[13] Dettori M., Riccardi N., Canetti D., Antonello R., Piana A., Palmieri A., Castiglia P., Azara A., Masia M.D., Porcu A. (2022). Infections in lung transplanted patients: A review. Pulmonology.

[14] Speich R., van der Bij W. (2001). Epidemiology and management of infections after lung transplantation. Clin. Infect. Dis..

[15] Dudau D., Camous J., Marchand S., Pilorge C., Rézaiguia-Delclaux S., Libert J., Fadel E., Stéphan F. (2014). Incidence of nosocomial pneumonia and risk of recurrence after antimicrobial therapy in critically ill lung and heart-lung transplant patients. Clin. Transplant..

[16] Boussaud V., Guillemain R., Grenet D., Coley N., Souilamas R., Bonnette P., Stern M. (2008). Clinical outcome following lung transplantation in patients with cystic fibrosis colonized with burkholderia cepacia complex: Results from two french centres. Thorax.

[17] Kumar A., Advani S., Asim K., Mohamed M.A., Wani F., Singh J., Albosta M., Shiwalkar N., Keshavamurthy S. (2022). Hyperammonemia in lung transplant patients and its management: A review. Indian J. Thorac. Cardiovasc. Surg..

[18] Mortensen E., Hellinger W., Keller C., Cowan L., Shaw T., Hwang S., Pegues D., Ahmedov S., Salfinger M., Bower W. (2014). Three cases of donor-derived pulmonary tuberculosis in lung transplant recipients and review of 12 previously reported cases: Opportunities for early diagnosis and prevention. Transpl. Infect. Dis..

[19] Friedman D.Z.P., Cervera C., Halloran K., Tyrrell G., Doucette K. (2020). Non-tuberculous mycobacteria in lung transplant recipients: Prevalence, risk factors, and impact on survival and chronic lung allograft dysfunction. Transpl. Infect. Dis..

[20] Daley C.L., Iaccarino J.M., Lange C., Cambau E., Wallace R.J., Andrejak C., Böttger E.C., Brozek J., Griffith D.E., Guglielmetti L. (2020). Treatment of nontuberculous mycobacterial pulmonary disease: An official ATS/ERS/ESCMID/IDSA clinical practice guideline. Eur. Respir. J..

[21] Hirama T., Singer L.G., Brode S.K., Marras T.K., Husain S. (2021). Treatment outcomes of nontuberculous mycobacterial pulmonary disease in lung transplant recipients. Transpl. Infect. Dis..

[22] Calder P.C., Carr A.C., Gombart A.F., Eggersdorfer M. (2020). Optimal nutritional status for a well-functioning immune system is an important factor to protect against viral infections. Nutrients.

[23] Kurihara C., Manerikar A., Querrey M., Felicelli C., Yeldandi A., Garza-Castillon R., Lung K., Kim S., Ho B., Tomic R. (2022). Clinical characteristics and outcomes of patients with COVID-19-associated acute respiratory distress syndrome who underwent lung transplant. JAMA.

[24] Humar A., Snydman D., AST Infectious Diseases Community of Practice (2009). Cytomegalovirus in solid organ transplant recipients. Am. J. Transplant..

[25] Kotton C.N., Huprikar S., Kumar D. (2017). Transplant infectious diseases: A review of the scientific registry of transplant recipients published data. Am. J. Transplant..

[26] Shah P.D., McDyer J.F. (2010). Viral infections in lung transplant recipients. Semin. Respir. Crit. Care Med..

[27] Danziger-Isakov L., Kumar D., AST ID Community of Practice (2019). Vaccination of solid organ transplant candidates and recipients: Guidelines from the american society of transplantation infectious diseases community of practice. Clin. Transplant..

[28] Walsh E.E., Marc G.P., Zareba A.M., Falsey A.R., Jiang Q., Patton M., Polack F.P., Llapur C., Doreski P.A., Ilangovan K. (2023). Efficacy and safety of a bivalent RSV prefusion F vaccine in older adults. N. Engl. J. Med..

[29] Griffiths C., Drews S.J., Marchant D.J. (2017). Respiratory syncytial virus: Infection, detection, and new options for prevention and treatment. Clin. Microbiol. Rev..

[30] Garegnani L., Styrmisdóttir L., Roson Rodriguez P., Escobar Liquitay C.M., Esteban I., Franco J.V. (2021). Palivizumab for preventing severe respiratory syncytial virus (RSV) infection in children. Cochrane Database Syst. Rev..

[31] Dimopoulos G., Almyroudi M., Myrianthefs P., Rello J. (2021). COVID-19-associated pulmonary aspergillosis (CAPA). J. Intensive Med..

[32] Singh N., Winston D.J., Razonable R.R., Lyon G.M., Silveira F.P., Wagener M.M., Stevens-Ayers T., Edmison B., Boeckh M., Limaye A.P. (2020). Effect of preemptive therapy vs antiviral prophylaxis on cytomegalovirus disease in seronegative liver transplant recipients with seropositive donors: A randomized clinical trial. JAMA.

[33] Uhlin M., Mattsson J., Maeurer M. (2012). Update on viral infections in lung transplantation. Curr. Opin. Pulm. Med..

[34] Hagerty J.A., Ortiz J., Reich D., Manzarbeitia C. (2003). Fungal infections in solid organ transplant patients. Surg. Infect. Larchmt.

[35] Phoompoung P., Villalobos A.P.C., Jain S., Foroutan F., Orchanian-Cheff A., Husain S. (2022). Risk factors of invasive fungal infections in lung transplant recipients: A systematic review and meta-analysis. J. Heart Lung Transplant..

[36] Marinelli T., Rotstein C. (2021). Invasive fungal infections in lung transplant recipients. Clin. Infect. Dis..

[37] Silveira F.P., Husain S. (2008). Fungal infections in lung transplant recipients. Curr. Opin. Pulm. Med..

[38] Pennington K.M., Dykhoff H.J., Yao X., Sangaralingham L.R., Shah N.D., Peters S.G., Barreto J.N., Razonable R.R., Kennedy C.C. (2021). The impact of antifungal prophylaxis in lung transplant recipients. Ann. Am. Thorac. Soc..

[39] Monforte A., Los-Arcos I., Martín-Gómez M.T., Campany-Herrero D., Sacanell J., Berastegui C., Márquez-Algaba E., Sempere A., Nuvials X., Deu M. (2022). Safety and effectiveness of isavuconazole treatment for fungal infections in solid organ transplant recipients (ISASOT study). Microbiol. Spectr..

[40] Wołyniec W., Sulima M., Renke M., Dębska-Ślizień A. (2018). Parasitic infections associated with unfavourable outcomes in transplant recipients. Medicina.

[41] Khurana S., Batra N. (2016). Toxoplasmosis in organ transplant recipients: Evaluation, implication, and prevention. Trop. Parasitol..

[42] Mobley C.M., Dhala A., Ghobrial R.M. (2017). Strongyloides stercoralis in solid organ transplantation: Early diagnosis gets the worm. Curr. Opin. Organ. Transplant..

[43] Backhus L.M., Mulligan M.S., Ha R., Shriki J.E., Mohammed T.H. (2016). Imaging in lung transplantation: Surgical considerations of donor and recipient. Radiol. Clin. N. Am..

[44] Liu H., Xu H., Liu H., Zhao Z., Zhang X. (2023). Metagenomic next-generation sequencing in the diagnose of pulmonary infection with airway complications in a lung transplant recipient. Heliyon.

[45] Veit T., Pan M., Munker D., Arnold P., Dick A., Kunze S., Meiser B., Schneider C., Michel S., Zoller M. (2021). Association of CMV-specific T-cell immunity and risk of CMV infection in lung transplant recipients. Clin. Transplant..

